# Supplementary material for: Identifying prognostic subgroups of luminal-A breast cancer using deep autoencoders and gene expressions
Source: PLoS Comput Biol. 2023 May 30;19(5):e1011197. doi: 10.1371/journal.pcbi.1011197 (PMC10256220; doi:10.1371/journal.pcbi.1011197)
Supplement: S1 Fig — (DOCX) [file pcbi.1011197.s001.docx]

**S1 Fig. The Kaplan-Meier survival analysis according to the dimensional size of latent features in METABRIC (the number of subgroups=2)**

| META-  BRIC | cluster1 | cluster2 |
| --- | --- | --- |
| # of samples | 282 | 397 |

**(a) The dimensional size = 16**

**
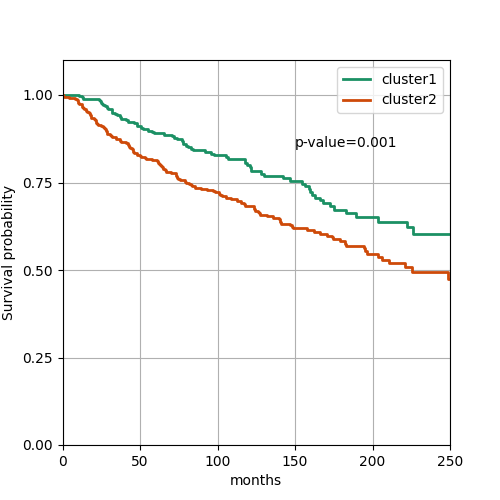
**

**(b) The dimensional size = 32**

**
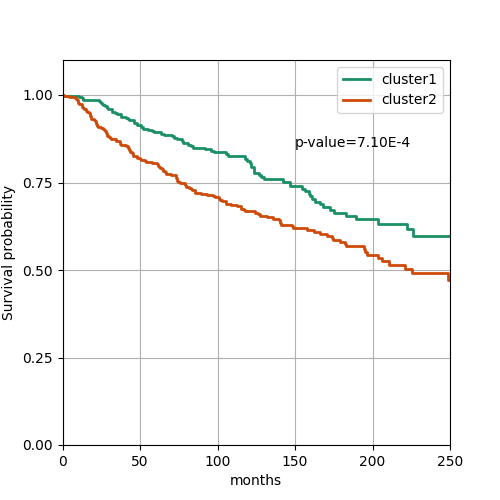
**

| META-  BRIC | cluster1 | cluster2 |
| --- | --- | --- |
| # of samples | 312 | 367 |

**(c) The dimensional size = 64**

| META-  BRIC | cluster1 | cluster2 |
| --- | --- | --- |
| # of samples | 336 | 343 |

**
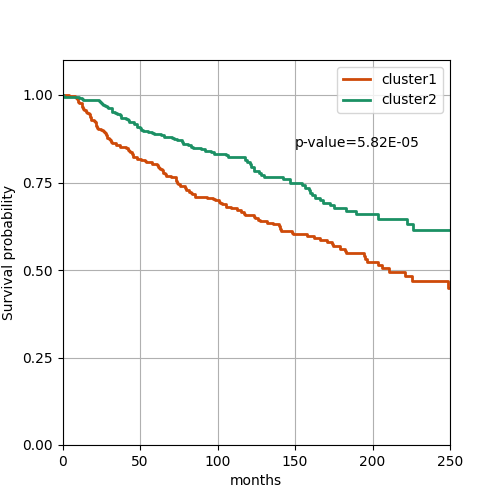
**

**(d) The dimensional size = 128**


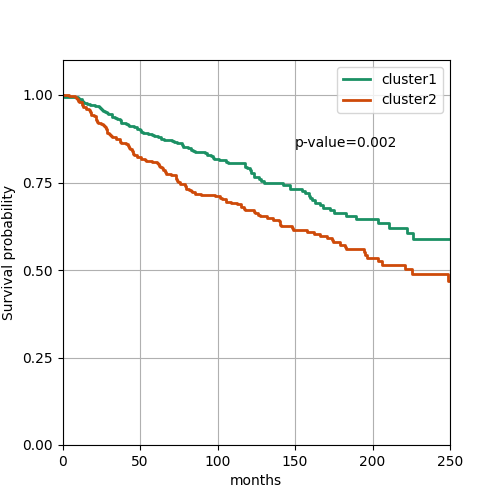


| META-  BRIC | cluster1 | cluster2 |
| --- | --- | --- |
| # of samples | 351 | 328 |
