# Supplementary material for: Identifying prognostic subgroups of luminal-A breast cancer using deep autoencoders and gene expressions
Source: PLoS Comput Biol. 2023 May 30;19(5):e1011197. doi: 10.1371/journal.pcbi.1011197 (PMC10256220; doi:10.1371/journal.pcbi.1011197)

**S2 Fig. The Kaplan-Meier survival analysis according to the number of clusters in the METABRIC dataset (64-dimensional latent features)**

**
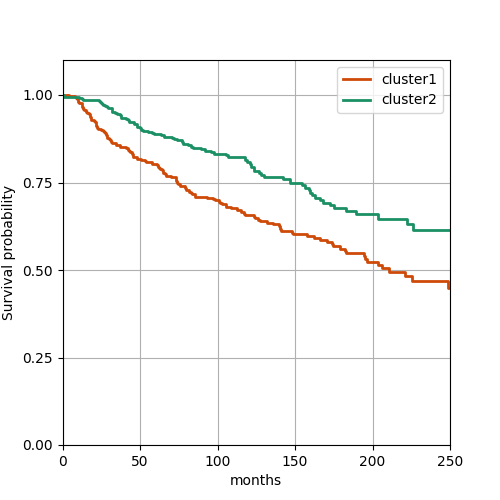
**(a) The cluster number=2 (b) The cluster number=3

**
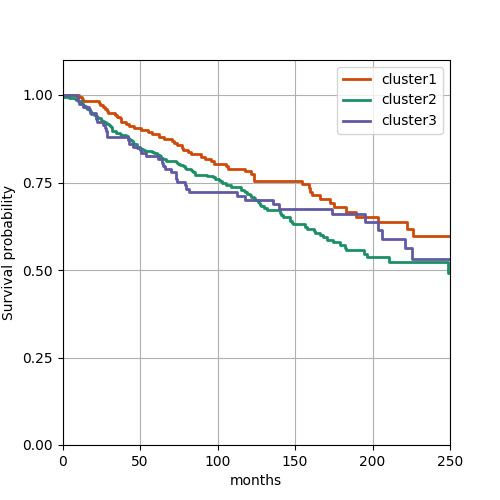
**

(a) The cluster number=4 (b) The cluster number=5


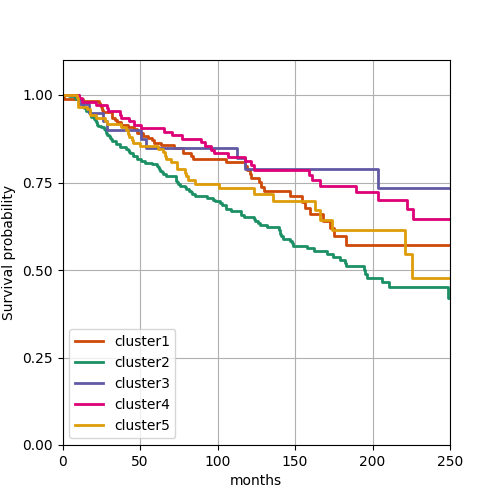

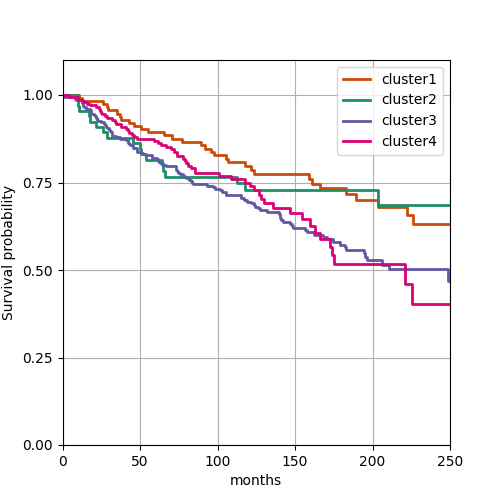

Supplement: S2 Fig — (DOCX) [file pcbi.1011197.s002.docx]
