# Supplementary material for: Identifying prognostic subgroups of luminal-A breast cancer using deep autoencoders and gene expressions
Source: PLoS Comput Biol. 2023 May 30;19(5):e1011197. doi: 10.1371/journal.pcbi.1011197 (PMC10256220; doi:10.1371/journal.pcbi.1011197)
Supplement: Attachment — Submitted filename: response_letter.pdf [file pcbi.1011197.s015.pdf]

Response Letter to Reviewers

Journal: PLOS Computational biology
Manuscript Number: PCOMPBIOL-D-23-00002
Article Type: Research article
Title: Identifying prognostic subgroups of luminal-A breast cancer using deep autoencoders and gene expressions
Authors: Seunghyun Wang, Doheon Lee

Dear Editors,

We appreciate your careful comments for reviewing our manuscript, titled “Identifying prognostic subgroups of luminal-A breast cancer using deep autoencoders and gene expressions”. We significantly improved our manuscripts based on the reviewer’s suggestions. Please find our point-by-point response letters below. The original comments are colored in black and our responses are highlighted in blue. We also provided modified texts with line numbers in the revised manuscript. Please do not hesitate to contact when you have further questions in the revised manuscript.

Sincerely,

Doheon Lee, Ph.D.

Professor, Dept. of Bio and Brain Engineering, KAIST, KOREA
Email: dhlee@kaist.ac.kr
Phone: +82-42-350-4316

On behalf of all authors.

Response to Reviewer #1

[Major comment 1] I'd like to recommend trying a combined approach of preliminary gene filtering and autoencoder, i.e. low variance gene filtering followed by autoencoder training. Here, I'd recommend selecting highly variable genes after variance stabilization to avoid the preference of highly expressed genes. If successful, it will help us to achieve a more compact model.

Response: Thank for your careful comment. As you mentioned, we chose the top 5,000 genes with the highest variability (**line 147-149, line 367**) to make a more compact model and we trained the deep autoencoders using these 5,000 genes. Furthermore, because the number of genes were decreased from 17,202 to 5,000, we additionally trained two more autoencoders of which the size of hidden layer is 16 and 32 (**line 150-151**). When we clustered the 679 samples of the METABRIC dataset using the 64-dimensional latent features and implemented the survival analysis, we confirmed that the two subgroups show more distinct prognostic difference (p-value=5.82E-05) compared to our previous model that uses whole genes (p-value=6.43E-04). We specifically described these results in **line 179-192**.

<u>Please find the corresponding text changed below:</u>

Line 147-149:

"Before training the deep autoencoders, we chose the top 5,000 genes which show the highest variability across the samples based on the median absolute deviation (MAD) in the METABRIC dataset (S1 Table)."

Line 367:

"we chose the top 5,000 genes with the highest variability based on the median absolute deviations of each gene."

Line 150-151:

"We trained the eight deep autoencoders with the different hidden layer size (16, 32, 64, 128, 256, 512, 1024, and 2048)."

Line 179-192:

"As a result, the prognostic differences between the subgroups were stably significant (p-value < 0.01) when the samples were divided into two subgroups regardless of the dimensional size of latent features (S1 Figure), but the difference was the most significant when the dimensional size is 64 (p-value=5.82E-05, Fig 2). However, when the samples were clustered into more than two subgroups using the 64-dimensional latent, and we found that some subgroup pairs did not show significant prognostic differences in the pairwise log-rank test. For example, when the samples were clustered into the three subgroups we observed that the samples belonged to the third subgroup (cluster3) did not show the significant prognostic differences with the samples belonged to the other subgroups (cluster1 and cluster2) (S3 Table and S2 Figure). We observed similar tendencies when we divided the samples into four and five subgroups (S3 Table and S2 Figure).
Given these results, we concluded that the prognostic differences were the most distinct when 679 METABRIC samples were divided into the two subgroups using the 64-dimensional latent features (Fig 2A). In the Kaplan-Meier survival curve (Fig 2B), the first subgroup (n=336) and the second subgroup (n=343) show worse and better prognosis (S4 Table). In the following sections of this study, we named the better prognostic subgroup s 'BPS-LumA', and the worse prognostic subgroup as 'WPS-LumA'."

[Major comment 2] On a second note, it is somewhat surprising to see that an autoencoder model trained using array data successfully classifies RNA-seq data. Is it because of the min-max normalization and highly correlated array vs RNA-seq data? Please discuss.

Response: Thank you for pointing this out. To further explore this result, we measured the Spearman's rank correlation coefficient (SRCC) of the mean expression levels in microarray (METABRIC) and RNA-seq (TCGA). In addition, we measured the SRCC of the absolute median deviations (MAD) of each gene in the both datasets. We observed that the mean expression levels of each gene are highly correlated (SRCC=0.771) in the both dataset and the MADs are also significantly correlated (SRCC=0.595). In addition to similar expression profiling results of the two platform, additional preprocessing to reduce the batch effects (e.g. min-max scaling) makes our methods successfully predicts the prognosis of the samples regardless of the expression profiling platforms. We described these results in **line 204-212** and **Fig. C-D**.

<u>Please find the corresponding text changed below:</u>

Line 204-212:

"Interestingly, even though the two datasets used the different expression profiling platforms (The METABRIC and TCGA dataset use microarray and RNA-seq as expression profiling platforms, respectively), we proved that our method is applicable in the both datasets. To further explore these results, we measured the Spearman's rank correlation coefficient (SRCC) between the mean expression levels of individual genes in the both datasets and we observed that they are highly correlated (SRCC=0.771, Fig 3C). Similarly, we measured the SRCC of the absolute median deviations of individual gene in the both datasets and we confirmed that they are also significantly correlated (SRCC=0.595, Fig 3D). In addition to these correlations between two expression profiling platforms, additional preprocessing to reduce the batch effects (e.g. min-max scaling) makes our methods successfully predicts the prognosis of the samples regardless of the expression profiling platforms."

[Major comment 3] Given the nature of WGCNA relying on correlation coefficient, it conveys a similar risk when involving low variance genes. How would the author justify this? What if similar preliminary gene filtering is applied?

Response: We appreciate your careful readings on the Method part. Because WGCNA provides the weights of every gene pair (fully connected), it requires a threshold to use only reliably correlated gene pairs. Therefore, we used only top 10% edges with the highest weights and their nodes in each module for the analysis but we found this unclearly stated in the Methods section. We now clearly described this in the Method section (**line 462-463**) of our revised manuscript.

<u>Please find the corresponding text changed below:</u>

Line 462-463:

"In addition, because WGCNA provides the weights of every gene pair (fully connected), we only used top 10% edges with the highest weights and their nodes in each module."

[Minor comment 1] In Fig 6, Panel B and C are not quite informative. Rather I'd recommend putting 1) a bar plot module size, and 2) % of genes in each module overlapping with DEG. Panels seem to be mislabeled in the legend for B and C. A appears twice, B once, and no C.

Response: We appreciate your considerate comments on our figures. As you mentioned, we plotted bar graphs that represent the module size and the proportion of genes overlapping with the DEGs in each module (**Fig. 5A-B**) In addition, we corrected the legend. (**line 285-288**)

Please find the corresponding text changed below:

Line 285-288:

“Fig 5. The module size and the proportion of genes overlapping with the DEGs in each co-expressed module
(A) The bar graph of the module size (the number of genes in each module). (B) The bar graph representing the % of genes overlapping with DEG. The lightcyan module is highlighted as yellow. (C) The network plot of the lightcyan module. The DEGs are colored as yellow and the node size indicates the connectivity of each node”

[Minor comment 2] In line 413, the authors described that the hidden layer size is 2048. However, they tried all different numbers of hidden layers in the supplementary figures. Please correct if necessary.

Response: Thank you for pointing this out. We concluded that the supplementary figures are not necessary and we removed the supplementary figures in our revised manuscript to avoid misunderstanding.

Response to Reviewer #2

[Major comment 1] While the use of autoencoder to extract potential latent features for stratifying breast cancer patients is reasonable, the proposed method lacks novelty. The authors mainly train a standard autoencoder with different latent dimensions using breast cancer gene expression data, which is technically not novel. Except for varying the dimension size, no other architecture/hyperparameter optimization is performed to customize the model for the task and enhance the stratification results.

Response: We appreciate your careful readings on our manuscript. We found the description of the novelty of our method unclearly stated in the manuscript. Even though our method used standard autoencoder models, our method has important biological contributions in realizing precision medicine of luminal-A breast cancer. First of all, to our best knowledge, it is the first attempt to use the deep autoencoders to discover the prognostic subgroups of luminal-A breast cancer (**line 164-168, line 179-192**). Moreover, we validated our result in the independent dataset, which indicates that our method is insensitive to expression profiling platform and batch effects **(line 196-203).** This is the most important point when the machine learning approach is translated into clinical practices. In addition, we showed that the latent features generated from the deep autoencoders are more useful that the gene expression profiles and the features generated from the traditional dimensionality reduction method (i.e. PCA) in terms of discovering the prognostic subgroups (**line 216-232**). Furthermore, we suggest biological mechanism that could be potentially determine the prognostic difference in luminal-A breast cancer and it can be a starting point to develop new drugs **(line 265-278).**

Please find the corresponding text changed below:

Line 164-168:
"The ultimate goal of our study is to identify distinct prognostic subgroups of luminal-A breast cancer. Hence, we clustered 679 METABRIC samples using the latent features generated from the deep autoencoders and unsupervised learning. We used K-means clustering for unsupervised learning and we set the cluster number as two to five. Then, we performed the Kaplan-Meier survival analysis to compare prognosis between the subgroups and the significance was evaluated by log-rank test."

Line 179-192:

"As a result, the prognostic differences between the subgroups were stably significant (p-value < 0.01) when the samples were divided into two subgroups regardless of the dimensional size of latent features (S1 Figure), but the difference was the most significant when the dimensional size is 64 (p-value=5.82E-05, Fig 2). However, when the samples were clustered into more than two subgroups using the 64-dimensional latent, and we found that some subgroup pairs did not show significant prognostic differences in the pairwise log-rank test. For example, when the samples were clustered into the three subgroups we observed that the samples belonged to the third subgroup (cluster3) did not show the significant prognostic differences with the samples belonged to the other subgroups (cluster1 and cluster2) (S3 Table and S2 Figure). We observed similar tendencies when we divided the samples into four and five subgroups (S3 Table and S2 Figure).
Given these results, we concluded that the prognostic differences were the most distinct when 679 METABRIC samples were divided into the two subgroups using the 64-dimensional latent features (Fig 2A). In the Kaplan-Meier survival curve (Fig 2B), the first subgroup (n=336) and the second subgroup (n=343) show worse and better prognosis (S4 Table). In the following sections of this study, we named the better prognostic subgroup s 'BPS-LumA', and the worse prognostic subgroup as 'WPS-LumA'."

Line 196-203:

"To validate the prognostic differences between the BPS-LumA and the WPS-LumA, we applied our stratification method to an independent dataset. We used the 415 TCGA luminal-A breast cancer samples for validation. We

generated the 64-dimensional latent features of each TCGA sample using the deep autoencoder trained with METABRIC dataset in the previous section, and each sample was assigned to the closer subgroup based on the distance between the sample and the centroid of the BPS-LumA and the WPS-LumA in the latent space (Fig 3A). Among 415 samples, 191 and 224 samples belonged to the BPS-LumA and the WPS-LumA, respectively (S5 Table). Interestingly, the samples belonging to the BPS-LumA showed significantly better prognosis than the other samples belonging to the WPS-LumA (p-value = 0.004; Fig 3B)."

Line 230-245:

"Next, we wanted to compare our method with gene expression profiles and traditional dimensionality reduction method to show the usefulness of the deep autoencoders in terms of generating the informative latent features for the prognostic subgroup identification. Hence, similarly to when using the latent features, we divided the 679 METABRIC samples into the two subgroups using the expression profiles of whole 17,202 genes and the top 5,000 genes with the highest variability as the input features of K-Means clustering, respectively. Interestingly, we found that the prognostic difference between the subgroups identified using the whole 17,202 genes (p-value = 0.566; Fig 4A) and the top 5,000 most variable genes (p-value = 0.183; Fig 4B) were not significant.

In addition, we compared our method with traditional dimensionality reduction method. To this end, we projected the whole 17,202 genes and the top 5,000 most variable genes into 64- (the same dimensional size with the latent features that we used) and 2- (the most informative principal components) dimensional space using PCA (Principal Component Analysis), respectively. As a result, we observed that none of them successfully discovered the distinct prognostic subgroups (Fig 4D-F).

From these results, we confirmed that only the latent features were able to identify the prognostic subgroups p-value=5.82E-05; Fig 2A). It indicates that the deep autoencoders more effectively extract the important properties from the complex gene expression profiles, which determine the prognosis of luminal-A breast cancer, than the traditional dimensionality reduction method and it helps to discover the distinct prognostic subgroups for precision medicine."

Line 268-282:

"As a result, we observed that "lightcyan" co-expressed module includes the significantly large number of DEGs (11.6%, Fig 5B and S8 Table). We explored the biological functions related to the lightcyan module through the gene-set enrichment analysis [26] and we observed that the 95 genes included in the lightcyan module was associated with ribosome-related terms such as "rRNA metabolic process (GO:0016072)", "Ribosome (MAP03010)", and "Cytoplasmic Ribosomal Proteins (WP477)" (S9 Table). Among the 95 genes in the lightcyan module, 11 genes were DEGs between BPS-LumA and WPS-LumA (Fig 5C).

We found that there are many literature evidences that report the associations between the ribosomal proteins (RPs) and cancer [27]. For example, the RP-MDM2-p53 signaling pathway is the most well-studied pathway which defines a role of ribosomal proteins in tumor suppressor gene p53 activation [28]. Recently, it was revealed that deregulation of some ribosomal proteins can promote breast cancer metastasis [29] and ribosome biogenesis could be potential therapeutic target to combat tamoxifen resistance [30]. Based on these results, the terms significantly associated with the DEGs and their activities in each subgroup could be considered as potential biological mechanisms that promote the prognostic differences between the BPS-LumA and WPS-lumA.."

[Major comment 2] The authors show that using the latent dimension of 2048 led to the best (most significant results). But considering that this latent dimension size was the largest among the test dimension sizes, and as this dimension size is comparable to the dimension of the original gene space, it is highly likely that the model is overfitted to the data (especially, since the sample size of the gene expression data was relatively small compared to the latent dimension).

Response: Thank for your careful comment. Unlike our original manuscript, we chose the top 5,000 genes with the highest variability (**line 147-149, line 367)** to make a more compact model and we trained the deep autoencoders using these 5,000 genes. Furthermore, because the number of genes were decreased from 17,202 to 5,000, we additionally trained two more autoencoders of which the size of hidden layer is 16 and 32 (**line 150-151**). We observed that the MSEs decreased until the size of hidden layers increases to 128. However, they increased again when the size of hidden layer is larger than 128, which means that the models are overfitted to the training set (**S2 Table**). Therefore, we decided to consider only the latent features obtained from the four models of which the size of hidden layer is 16, 32, 64, and 128 (**line 156-163**). We finally decided to use 64-dimensional latent features which are not only relatively small than the sample size but also show the most distinct prognostic difference in the following analysis (**line 179-192**)

Please find the corresponding text changed below:

Line 147-149:

"Before training the deep autoencoders, we chose the top 5,000 genes which show the highest variability across the samples based on the median absolute deviation (MAD) in the METABRIC dataset (S1 Table)."

Line 367:

"we chose the top 5,000 genes with the highest variability based on the median absolute deviations of each gene."

Line 150-151:

"We trained the eight deep autoencoders with the different hidden layer size (16, 32, 64, 128, 256, 512, 1024, and 2048)."

Line 156-163:

"As we expected, the MSEs decreased continuously as the size of hidden layers increased in the training set (S2 Table), but the differences between the deep autoencoders were not that significant (MSE=0.012\pm0.008). In the validation set, we observed that the differences between the deep autoencoders were much smaller than the training set (MSE=0.075\pm0.003). More importantly, the MSEs decreased until the size of hidden layers increased to 128. However, they started to increase again from when the size of hidden layer is larger than 128. It indicates that the models might be overfitted to the training set. Therefore, we decided to use only the latent features obtained from the models of which the size of hidden layers are 16, 32, 64, and 128 in the following survival analysis."

Line 179-192:

"As a result, the prognostic differences between the subgroups were stably significant (p-value < 0.01) when the samples were divided into two subgroups regardless of the dimensional size of latent features (S1 Figure), but the difference was the most significant when the dimensional size is 64 (p-value=5.82E-05, Fig 2). However, when

the samples were clustered into more than two subgroups using the 64-dimensional latent, and we found that some subgroup pairs did not show significant prognostic differences in the pairwise log-rank test. For example, when the samples were clustered into the three subgroups we observed that the samples belonged to the third subgroup (cluster3) did not show the significant prognostic differences with the samples belonged to the other subgroups (cluster1 and cluster2) (S3 Table and S2 Figure). We observed similar tendencies when we divided the samples into four and five subgroups (S3 Table and S2 Figure).
Given these results, we concluded that the prognostic differences were the most distinct when 679 METABRIC samples were divided into the two subgroups using the 64-dimensional latent features (Fig 2A). In the Kaplan-Meier survival curve (Fig 2B), the first subgroup (n=336) and the second subgroup (n=343) show worse and better prognosis (S4 Table). In the following sections of this study, we named the better prognostic subgroup s 'BPS-LumA', and the worse prognostic subgroup as 'WPS-LumA'."

[Major comment 3] The latent features do not seem to have any biological significance. For example, while there has been recent work on constraining the latent space based on pathway info - in which case the latent features may potentially reflect underlying pathway activities, in this current work it is difficult to see whether the 2,048 latent features meaningfully reflect the underlying biological processes/pathways that may be associated with different prognosis of subgroups. Although the authors perform some analysis by analyzing the differentially expressed genes between the subgroups that are identified by the proposed method, such analysis beats the purpose of using an autoencoder for latent feature extraction in the first place. For example, we could have simply started with DEG analysis without using autoencoders to detect gene markers that can be used to stratify subgroups and then analysis the identified DEGs to understand what might be leading to this difference. Why should one first use autoencoders to extract latent features (that cannot be easily interpreted), and then resort to DEG analysis for interpretation?

Response: Thank for pointing this out. While we appreciate your comment, we confirmed that the two subgroups, which are clustered using the whole genes and top 5,000 genes with the highest variability, do not show significant prognostic difference (**Fig 4A and Fig 4B, respectively**) (**line 216-222**). Therefore, we concluded that it is meaningless to find out DEGs between them to predict the prognosis.
Instead, we used a traditional individual-gene based approach, which is the most frequent to find the prognostic marker genes. We divided the 679 METABRIC samples into two subgroups based on the median expression levels (re-normalized gene expression profiles using min-max scaling) of every individual gene. As a result, among 17,202 genes, we observed that the 589 genes discover the significantly distinct prognostic subgroups (p-value < 0.01). However, when the same criteria (median gene expression value of each gene) is applied to the 415 TCGA samples, we confirmed that the prognostic differences are significant in only 9 out of the 589 genes (1.53%) and none of them is more distinct than when the latent features generated from the autoencoder are used (p-value=5.82E-05). In addition, we implemented pathway enrichment analysis using these 9 genes and we found that they are not significantly associated with any biological pathways. These results indicate that the traditional individual gene-based approach is very sensitive to the expression profiling platform and batch effects and it makes it difficult to stably predict the prognosis in independent datasets. We did not include this part in our revised manuscript because we fully described the results about the comparison with other features (features generated from traditional dimensionality reduction method, i.e., PCA) (**line 223-227**).
However, we showed that our method successfully discovers the prognostic subgroups in the independent dataset (**line 196-203**). In addition, our DEG analysis using co-expression network of breast tissue successfully found the relevant biological functions associated with the DEGs between the two subgroups (**line 266-278**). Furthermore, the recent efforts to develop explainable and interpretable deep learning models could help interpret the biological meanings of the latent features and we mentioned it in the Discussion section. (**line 335-337**).

Please find the corresponding text changed below:

Line 216-222:

"Next, we wanted to compare our method with gene expression profiles and traditional dimensionality reduction method to show the usefulness of the deep autoencoders in terms of generating the informative latent features for

the prognostic subgroup identification. Hence, similarly to when using the latent features, we divided the 679 METABRIC samples into the two subgroups using the expression profiles of whole 17,202 genes and the top 5,000 genes with the highest variability as the input features of K-Means clustering, respectively. Interestingly, we found that the prognostic difference between the subgroups identified using the whole 17,202 genes (p-value = 0.566; Fig 4A) and the top 5,000 most variable genes (p-value = 0.183; Fig 4B) were not significant."

Line 223-227:

"In addition, we compared our method with traditional dimensionality reduction method. To this end, we projected the whole 17,202 genes and the top 5,000 most variable genes into 64- (the same dimensional size with the latent features that we used) and 2- (the most informative principal components) dimensional space using PCA (Principal Component Analysis), respectively. As a result, we observed that none of them successfully discovered the distinct prognostic subgroups (Fig 4D-F)."

Line 196-203:

"To validate the prognostic differences between the BPS-LumA and the WPS-LumA, we applied our stratification method to an independent dataset. We used the 415 TCGA luminal-A breast cancer samples for validation. We generated the 64-dimensional latent features of each TCGA sample using the deep autoencoder trained with METABRIC dataset in the previous section, and each sample was assigned to the closer subgroup based on the distance between the sample and the centroid of the BPS-LumA and the WPS-LumA in the latent space (Fig 3A). Among 415 samples, 191 and 224 samples belonged to the BPS-LumA and the WPS-LumA, respectively (S5 Table). Interestingly, the samples belonging to the BPS-LumA showed significantly better prognosis than the other samples belonging to the WPS-LumA (p-value = 0.004; Fig 3B)."

Line 266-278:

"As a result, we observed that "lightcyan" co-expressed module includes the significantly large number of DEGs (11.6%, Fig 5B and S8 Table). We explored the biological functions related to the lightcyan module through the gene-set enrichment analysis [26] and we observed that the 95 genes included in the lightcyan module was associated with ribosome-related terms such as "rRNA metabolic process (GO:0016072)", "Ribosome (MAP03010)", and "Cytoplasmic Ribosomal Proteins (WP477)" (S9 Table). Among the 95 genes in the lightcyan module, 11 genes were DEGs between BPS-LumA and WPS-LumA (Fig 5C).
We found that there are many literature evidences that report the associations between the ribosomal proteins (RPs) and cancer [27]. For example, the RP-MDM2-p53 signaling pathway is the most well-studied pathway which defines a role of ribosomal proteins in tumor suppressor gene p53 activation [28]. Recently, it was revealed that deregulation of some ribosomal proteins can promote breast cancer metastasis [29] and ribosome biogenesis could be potential therapeutic target to combat tamoxifen resistance [30]. Based on these results, the terms significantly associated with the DEGs and their activities in each subgroup could be considered as potential biological mechanisms that promote the prognostic differences between the BPS-LumA and WPS-lumA."

Line 335-357:

"Furthermore, there are recently lots of efforts to develop explainable and interpretable deep leaning models to overcome "black-box" problem [33, 34], especially for biomedical and healthcare domain [35, 36]. These efforts could be helpful to interpret the biological meanings of the latent features generated in our method."

[Major comment 4] Figure 2 and Figure 3 show PCA plots for the latent feature vectors, but how does it compare to using PCA directly to gene expression data? Why not use PCA to extract important principal components that reflect the main gene expression patterns, use these principal components for clustering patients and stratifying them? Does the use of autoencoder significantly improve the results? Such comparison would be critical in demonstrating the effectiveness of the proposed scheme.

Response: We appreciate your careful readings on our manuscript. As you mentioned, we extracted 64- and 2-dimensional features from the whole gene expressions (N=17,202) and the top 5,000 genes with the highest variability using PCA. Then, we divided the samples into two subgroups and compared the prognosis. As a result, we observed that none of them successfully discovered the distinct prognostic subgroups (Fig 4D-F). These results indicate that the deep autoencoders more effectively extract the informative features from the complex gene expression profiles, which determine the prognosis of luminal-A breast cancer, than PCA. We specifically described this part in our revised manuscript (**line 223-232**).

<u>**Please find the corresponding text changed below:**</u>

Line 223-232:

"In addition, we compared our method with traditional dimensionality reduction method. To this end, we projected the whole 17,202 genes and the top 5,000 most variable genes into 64- (the same dimensional size with the latent features that we used) and 2- (the most informative principal components) dimensional space using PCA (Principal Component Analysis), respectively. As a result, we observed that none of them successfully discovered the distinct prognostic subgroups (Fig 4D-F).
From these results, we confirmed that only the latent features were able to identify the prognostic subgroups p-value=5.82E-05; Fig 2A). It indicates that the deep autoencoders more effectively extract the important properties from the complex gene expression profiles, which determine the prognosis of luminal-A breast cancer, than the traditional dimensionality reduction method and it helps to discover the distinct prognostic subgroups for precision medicine."

[Major comment 5] Furthermore, there have been pathway-based methods and network analysis-based methods for extracting gene modules that may be used as diagnostic and/or prognostic markers. How does the use of autoencoder-based data driven scheme for extracting latent features compare with more traditional methods that define/predict modular markers based on pathways or network info? Is there a clear evidence that even without using such additional information (i.e., pathway info or network info), the autoencoder can extract better prognostic signatures there are predictive of patients' survival?

Response: Thank for pointing this out. The pathway-based methods and network-based methods could be used to find prognostic markers, but we concluded that they have some limitations. First of all, they are highly depending on prior knowledges, such as pre-defined pathways and known physical and functional interactions between genes. However, our knowledge about human interactome is still incomplete (Menche et al., Science, 2015 / Ghiassian et al., PLOS Computational biology, 2015). In addition, networks derived from experimental assays (e.g. Yeast two-hybrid) and computational inference usually do not fully consider the context-specificity (Anirudh Prahallad et al., Nature, 2012 / Joshoua Broyde et al., Nature Biotechnology, 2021). However, our method handles very specific subgroups of breast cancer. Therefore, we concluded that the deep autoencoders are more proper in terms of extracting the informative latent features from the gene expression profiles of luminal-A breast cancer, regardless of prior knowledge.

[Major comment 6] The authors claim that "our method and previous methods can be complementary to each other for a more precise stratification for luminal-A breast cancer". However, this conclusion is drawn simply based on the fact the difference/discrepancies between the different methods. To justify such statement, the authors should actually show "how" they could be combined to improve the stratification results.

Response: We appreciate your considerate comments on our study. We found that this claim is more suitable to the Discussion section and we moved them to the Discussion section. Moreover, we suggested a more specific and possible scenario of complementary uses of our and previous works with an example in the Discussion section (**line 338-345**).

Please find the corresponding text changed below:

Line 338-345:

"In addition, we showed that our and the previous methods are significantly coincident and they could complement each other for a more precise stratification through further study. For example, in the Poudel's method, the samples belonged to goblet-like subgroups are regarded as intermediate prognosis, which are ambiguous than the other subgroups. Interestingly, we discovered that 41.18% and 58.82% of them are belonged to the BPS-LumA and WPS-LumA in our method. This result indicates that even though the samples show similar goblet cell-like signatures, they could show different characteristics in other biological pathways, such as ribosome-related pathways that we discovered with the DEG analysis. In this perspective, comprehensively considering the results of our and the previous methods can be helpful to more precisely define the disease states of the patients."

[Minor comment 1] The literary presentation of the paper should be improved, as the current manuscript includes a large number of grammatical and typographical errors.

Response: Thank you for your comment. We tried to our best to correct the grammatical and typographical errors and more clearly described what we have done and found in our revised manuscript.

[Minor comment 2] In line 198, the author mentions that "the two datasets used the different sequencing platform", but note that microarray is not a "sequencing platform".

Response: Thank you for pointing this out. We changed the "sequencing platform" to "expression profiling platform" (**line 204-206**).

Please find the corresponding text changed below:

Line 204-206:

"Interestingly, even though the two datasets used the different expression profiling platforms (The METABRIC and TCGA dataset use microarray and RNA-seq as expression profiling platforms, respectively), we proved that our method is applicable in the both datasets."
