# Supplementary material for: Identifying prognostic subgroups of luminal-A breast cancer using deep autoencoders and gene expressions
Source: PLoS Comput Biol. 2023 May 30;19(5):e1011197. doi: 10.1371/journal.pcbi.1011197 (PMC10256220; doi:10.1371/journal.pcbi.1011197)
Supplement: Attachment — Submitted filename: response_letter.docx [file pcbi.1011197.s016.docx]

**Response Letter to Reviewers**

Journal: PLOS Computational biology

Manuscript Number: PCOMPBIOL-D-23-00002

Article Type: Research article

Title: Identifying prognostic subgroups of luminal-A breast cancer using deep autoencoders and gene expressions
Authors: Seunghyun Wang, Doheon Lee

Dear Editors,

We appreciate your careful comments for reviewing our revised manuscript and the responses, titled “Identifying prognostic subgroups of luminal-A breast cancer using deep autoencoders and gene expressions”. We significantly improved our manuscripts again based on the reviewer’s suggestions. Please find our point-by-point response letters below. The original comments are colored in black and our responses are highlighted in blue. We also provided modified texts with line numbers in the revised manuscript. Please do not hesitate to contact when you have further questions in the revised manuscript.

Sincerely,

Doheon Lee, Ph.D.

Professor, Dept. of Bio and Brain Engineering, KAIST, KOREA

Email: [dhlee@kaist.ac.kr](mailto:dhlee@kaist.ac.kr)

Phone: +82-42-350-4316

On behalf of all authors.

**Response to Reviewer #1**

**[Minor comment 1]** Authors changed the presentation of the dimension reduction in the main figures. What was the basis of this change.

**Response:** We appreciate your careful readings on our manuscript. The purpose of Fig. 2A and Fig. 3A was to visualize the positions of latent features of samples belonging to each prognostic subgroup in the latent space, so that they can be easily perceived by human visions. Based on this purpose, we chose t-SNE (t-distributed Stochastic Neighbor Embedding), because it was specifically designed for the purpose of visualization by preserving local neighbor structures and non-linearity of the data (Matten et al., Journal of Machine Learning Research, 2008). For this reason, t-SNE. For this reason, the t-SNE reduces the dimensionality of the data to only 2- or 3-dimensions.

On the other hand, PCA (Principal component analysis) more focus on the data dimension reduction preserving the property of original data and the data can be projected to larger dimensional size space. Therefore, in our revised manuscript, we added the task that compare the prognostic difference between two subgroups discovered by using the latent features generated by the deep autoencoders features and the features generated by PCA (Principal component analysis), (64-dimsional and 2-dimesional features) (**line 224-228**).

Therefore, we chose t-SNE to emphasize the purpose of the visualization in Fig. 2A and Fig. 3A and to avoid any misunderstanding in the additional task.

**Please find the corresponding text changed below:**

**Line 224-228:**

“In addition, we compared our method with traditional dimensionality reduction method. To this end, we projected the whole 17,202 genes and the top 5,000 most variable genes into 64- (the same dimensional size with the latent features that we used) and 2- (the most informative principal components) dimensional space using PCA (Principal Component Analysis), respectively. As a result, we observed that none of them successfully discovered the distinct prognostic subgroups (Fig 4D-F).”

**Response to Reviewer #2**

**[Minor comment 1]** Regarding my previous comment concerning the comparison against existing methods that used prior knowledge (e.g., pathways or PPI networks), while I understand the authors position I nevertheless think that it would be meaningful to include at least some discussion comparing the pros and cons of the respective approaches.

**Response:** Thank for your careful comment. Based on your comments, we concluded that the prior knowledges of network-based and pathway-based method could be helpful to increase the interpretability of deep learning models (e.g., visible neural network). We additionally described this part in the Discussion section of our revised manuscript **(line 339-345).**

**Please find the corresponding text changed below:**

**Line 339-345:**

“Alternatively, even though network-based or pathway-based methods have some limitations that our knowledge about human interactome is still incomplete [37, 38] and the interaction information derived from experimental assays (e.g. yeast two-hybrid) and computational inference usually do not fully consider the context-specificity [39, 40], they could suggest more solid evidences to interpret deep learning approaches when the prior knowledges are combined with the deep learning models (e.g., visible neural network [41]). Likewise, the approaches to increase the interpretability of deep learning models could accelerate the development of novel therapeutic options for the patients of poor prognosis.”

**[Minor comment 2]** Finally, the authors have revised a number of places in the manuscript to convey the novel contributions made in this work more clearly, these are spread over the paper and it may be a good idea to summarize the main novel contributions more clearly at the outset (e.g., in the last paragraph of the introduction section).

**Response:** We appreciate your careful readings on our manuscript. As you mentioned, we found that the descriptions of our contributions are spread over the paper and we stated our contributions more collectively in the last paragraph of the introduction section in our revised manuscript **(line 111-121).**

**Please find the corresponding text changed below:**

**Line 164-168:**

“In this study, we identified two prognostic subgroups of luminal-A breast cancer. To this end, we trained deep autoencoders using gene expression profiles of luminal-A breast cancer to generate informative latent features of each sample and we discovered the subgroups through the latent features and unsupervised learning. Additionally, we showed that our method has important biological contributions in realizing precision medicine of luminal-A breast cancer. First, we demonstrated that our method is feasible in an independent test set, which is the most important part to translate the deep learning approaches to clinical practices. Furthermore, we proved that the latent features are more useful than gene expression profiles and features generated by traditional dimensionality reduction method (i.e., PCA) in terms of discovering the prognostic subgroups. In addition, we suggest potential molecular mechanisms which determine the prognostic difference using differentially expressed genes between the subgroups and weighted gene co-expression network analysis. Lastly, we compared our stratification with two previous luminal-A breast cancer stratification methods.”
